# The Growth Characteristics and the Active Compounds of *Cudrania tricuspidata* Fruits in Different Cultivation Environments in South Korea

**DOI:** 10.3390/plants12112107

**Published:** 2023-05-25

**Authors:** Dong-Hwan Lee, Yong-Hwan Son, Jun-Hyuk Jang, Sun-Young Lee, Hyun-Jun Kim

**Affiliations:** Forest Medicinal Resources Research Center, National Institute of Forest Science, Yeongju-si 36040, Republic of Korea; leedh0419@korea.kr (D.-H.L.); thsdydghks@korea.kr (Y.-H.S.); wnseldu123@korea.kr (J.-H.J.); nararawood@korea.kr (S.-Y.L.)

**Keywords:** active compounds, correlation analysis, *Cudrania tricuspidata*, growth characteristics, method validation, UPLC-UV

## Abstract

*Cudrania tricuspidata* is a traditional medicinal herb in East Asia. The compounds of plants vary depending on environmental factors, such as soil, temperature, drainage, and so on. However, few to no studies have been done on the correlation among environment, growth, and compounds in *C. tricuspidata*. Thus, we aimed to investigate their relationship. Samples of *C. tricuspidata* fruit and cultivation soil were collected from 28 cultivation sites in October 2021. Six growth characteristics, eleven soil physicochemical properties, seven meteorological data points, and three active compounds were investigated in this study. We developed and validated an optimized method for quantifying active compounds using UPLC and performed correlation analysis of the environment, growth characteristics, and active compounds. The UPLC-UV method for determining active compounds was validated by measuring the linearity, LOD, LOQ, precision, and accuracy using UPLC. The LOD and LOQ were 0.01–0.03 µg/mL and 0.04–0.09 µg/mL, respectively. The precision was acceptable with RSD% values less than 2%. The recoveries ranged from 97.25 to 104.98% with RSD values <2%, within the acceptable limits. The active compounds were negatively correlated with the size of the fruit, and the growth characteristics were negatively correlated with some environmental factors. The results of this study can be used as basic data for the standard cultural practices and quality control of *C. tricuspidata* fruits.

## 1. Introduction

*Cudrania tricuspidata* is a deciduous broad-leaved thorny tree belonging to the Moraceae family and is widely distributed throughout East Asia, including Korea, China, and Japan [1]. It is known as cudrang, mandarin melon berry, silkworm thorn, and hariguwa (in Japanese) [2,3]. *C. tricuspidata* blooms in June and ripens between September and October. The bark of *C. tricuspidata* is grayish brown and has thorns whose length ranges from 0.5 to 3.5 cm. In addition, leaves of *C. tricuspidata* are split into three or have flat edges and are egg-shaped, both of which are found on a single tree [4]. In Eastern countries, *C. tricuspidata* has been used for traditional herbal medicines. In the Korean classic “Donguibogam”, it was reported that the fruit, leaf, and root of *C. tricuspidata* were used for deafness due to “Pungheo” and malaria [5]. In China, the roots of *C. tricuspidata* have been used for the treatment of bruising, gonorrhea, boils, rheumatism, scabies, jaundice, and dysmenorrhea in traditional Chinese medicine ‘Chuan-po-shi’ and the root bark has been used for the treatment of hemoptysis, contusions, and lumbago [6]. In addition, *C. tricuspidata* has been used in the treatment of eczema, mumps, tuberculosis, and acute arthritis in the Ancient Orient [7].

Over the last few decades, various bioactive properties of *C. tricuspidate* have been studied. Various physiological functions, such as anticancer [8,9,10,11,12,13,14], antioxidant [7,15,16,17,18], anti-inflammatory [19,20], anti-obesity [21,22,23], and antibacterial [24,25], etc., have been reported as part of the efficacy of *C. tricuspidata*. Due to these effects, the fruits of *C. tricuspidata* are used as fresh fruits, juice, jams, and alcoholic beverages, such as wine or vinegar [26]. These physiological functions are due to numerous secondary metabolites in plants. The secondary metabolites not only play an important role in the phytochemical defense of species against herbivores and insects but are also involved in non-biological stresses, such as temperature, light, precipitation, and inorganic nutrients [27,28]. 

Plants have the ability to produce a variety of metabolites, and over 1,000,000 metabolites are predicted to be present in the entire plant kingdom [29]. Flavonoids are one of the major groups of specialized metabolites and include over 9000 compounds [30]. The term “flavonoid” is used only for compounds with a C_6_-C_3_-C_6_ carbon framework that exhibits the structure of a chromane or that of a chromene, such as flavans, flavones, flanovols, and anthocyanidins [31]. Flavonoids in plants play important roles, such as pigments that attract pollinators, auxins regulation, and protection against stresses, such as ultraviolet-B, temperature, and heavy metals [32]. A variety of bioactive compounds, such as xanthones, flavonoids, organic acids, phenylpropanoids, and others, have been isolated from leaves, roots, fruits, twigs, and stems of *C. tricuspidata* [6].

The major compositions of the *C. tricuspidata* fruits have been identified as prenylated isoflavonoids, such as 6,8-diprenylorobol, 6,8-diprenylgenistein, and 4′-*O*-methylalpinumisoflavone [33]. It has been reported that 6,8-diprenylorobol has anti-obesity [6], anticancer [6], and neuroprotective [6,21] properties; 6,8-diprenylgenistein has anti-obesity [22], anti-inflammatory, antioxidant [6], and antibacterial [25] properties; and 4′-*O*-methylalpinumisoflavone has antioxidant [6], anti-inflammatory [34], and antitumor [35] properties. To use these bioactive compounds in industry, increasing the content of active compounds and standardizing the cultivation method are necessary.

Studies in which environmental factors affect the content of active compounds in plants have been reported [36,37,38]. The contents of active compounds vary depending on the plant’s growth characteristics, and the growth characteristics are affected by the growth environment [39]. Studies on the environmental factors affecting the growth characteristics or contents of active compounds are needed to improve the quality of *C. tricuspidata* fruits for use as functional raw materials. However, to our knowledge, no studies have been reported on the relationship among the compositions, growth, and environment of *C. tricuspidata* fruits. In this study, we aimed to investigate the correlation among active compounds, the growth characteristics of *C. tricuspidata* fruits, and environmental factors in different cultivation sites.

## 2. Results

### 2.1. Soil Characteristics and Meteorological Factors 

The eleven soil physicochemical properties were analyzed (Table 1). The results of soil texture analysis confirmed that it was sandy loam, except for two sites (site 2 and 22; loam). In the case of soil acidity, the highest was seen at site 11 at pH 6.75 ± 0.34, and the lowest was seen at site 20 at pH 4.21 ± 0.05. Only six sites belonged to the suitable acidity range (pH 5.5–6.5) for tree growth, and the value of electric conductivity was suitable at all sites [40]. The content of organic matter, total nitrogen, and cation exchange capacity were significantly higher at site 24 than at other sites. The content of available phosphate was highest at site 23 at 1105.91 ± 118.28 mg/kg. In the case of exchangeable cation, the highest Ca^2+^ was seen at site 11 at 16.84 ± 2.81 cmol^+^/kg and the lowest at site 19 at 0.76 ± 0.22 cmol^+^/kg. The highest Mg^2+^ was at site 14 at 3.18 ± 0.29 cmol^+^/kg and the lowest at site 6 at 0.11 ± 0.01 cmol^+^/kg. The value of base saturation was lowest at site 6 at 8.12 ± 1.42% and the highest at site 14 at 101.74 ± 0.20%. 

The meteorological data were collected for six factors (Table S1). The annual mean temperature was in the range of 11.4–15.6 °C. The annual mean maximum temperature and annual maximum temperature were highest at site 4 (21.1 °C, 37.8 °C). The annual mean minimum temperature was lowest at site 10 at 4.6 °C, and the annual minimum temperature was lowest at site 25 at −24.6 °C. The values of total precipitation were highest at site 5 at 1800.1 mm and lowest at site 1 at 923.0 mm.

### 2.2. Growth Characteristics

The growth characteristics data of *C. tricuspidata* fruits are shown in Table 2. The Gochang area (site 21) showed the highest average length and width of individual fruits, which were 33.25 ± 1.53 and 38.94 ± 2.84 mm, respectively, while the Inje area (site 1) showed the lowest value at 19.53 ± 0.37 and 23.66 ± 0.67 mm. The highest average fresh weight was 25.29 ± 1.47 g in the Sunchang area (site 23), while the lowest value was shown at 7.53 ± 0.72 g in the Hadong area. The sugar contents of fruit were of the highest average value in the Hadong area (site 6), which consisted of 19.40 ± 1.10 Brix°, whereas the Hampyeong area (site 18) showed the lowest value at 9.47 ± 0.33 Brix°. In addition, the Hwasun area (site 20) showed the greatest number of seeds, which was 32.67 ± 3.10, while the Hadong area (site 6) showed the lowest value at 9.33 ± 0.51. Moreover, the Yangsan area (site 4) had the highest aspect ratio of 1.29 ± 0.07, while the Cheonan area (site 27) had the lowest value at 1.08 ± 0.01. It was found that there was a negative correlation between the sugar contents of fruit and fruit size (length, width, fresh weight of fruit), and also a negative correlation between the sugar contents of fruit and the number of seeds (*p* < 0.01, Table S2).

### 2.3. Validtaion and Quantitative Analysis of Active Compounds 

The established UPLC method was also validated to assure its suitability for the analyses. The validation results are shown in Table 3, Table 4 and Table 5. They showed a good linear correlation coefficient (*r*^2^) for all calibration curves, which was higher than 0.999 for all analytes, indicating good linearity. Limits of detection (LOD) and quantification (LOQ) values of three active compounds (6,8-diprenylorobol, 6,8-diprenylgenistein, and 4′-*O*-methylalpinumisoflavone) were confirmed at 0.01 and 0.05 µg/mL, 0.03 and 0.09 µg/mL, and 0.01 and 0.04 µg/mL, respectively (Table 3). The intra-and inter-day variations were calculated by measuring the amounts of the three active compounds at three different concentration levels in triplicates. RSD values were calculated by using Equation (5), which ranged between 0.01% and 0.56% (intra-day) and between 0.20 and 1.82% (inter-day), which is within acceptable limits (Table 4). The recovery of the active compounds was calculated from Equation (6) to be 97.25–104.98%, and an RSD of <1.5% for accuracy was established by using Equation (5) (Table 5). These results validate the analytical method developed for the quality control of *C. tricuspidata* fruits using UPLC. 

**Figure 1:**
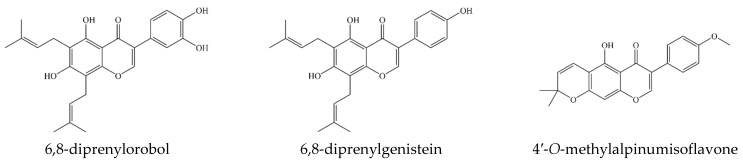
Structure of active compounds in *Cudrania tricuspidata* fruit.

**Figure 2:**
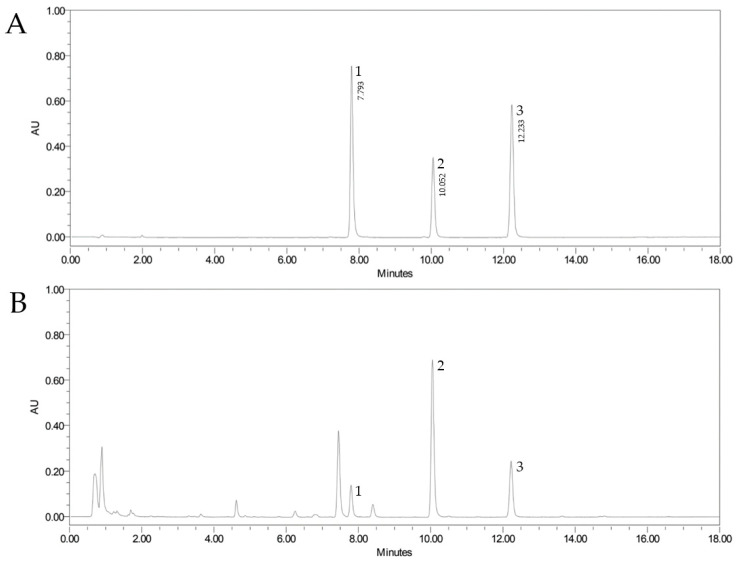
UPLC chromatogram of active compounds of the standard mixture (**A**) and *Cudrania tricuspidata* sample (**B**). 6,8-diprenylorobol (1), 6,8-diprenylgenistein (2), and 4′-*O*-methylalpinumisoflavone (3).

The UPLC-UV method was applied to the 133 samples of *C. tricuspidata* fruit. The active compounds were identified by comparing UV spectra chromatograms of the peaks and retention time with those of the standards in the UPLC-UV chromatogram. The three active compounds (6,8-diprenylorobol, 6,8-diprenylgenistein, and 4′-*O*-methylalpinumisoflavone) were detected at a retention time of 7.79, 10.05, and 12.23 min, respectively (Figure 1 and Figure 2). The quantitative results are shown in Table 6. The highest number of samples contained 6,8-diprenylorobol from 6.37 ± 0.63 to 137.55 ± 47.73 µg/g, 6,8-diprenylgenistein from 19.89 ± 0.78 to 294.68 ± 17.97 µg/g, and 4′-*O*-methylalpinumisoflavone from 10.61 ± 2.14 to 322.6 ± 11.9 µg/g. The highest content of 6,8-diprenylorobol was confirmed at cultivation site 1, of 6,8-diprenylgenistein at cultivation site 11, and of 4′-*O*-methylalpinumisoflavone at cultivation site 16. The total content of active compounds was confirmed to be 647.14 ± 61.06 µg/g at cultivation site 1.

### 2.4. Correlation Analysis 

Correlation analysis between growth characteristics and environmental data (soil characteristics and meteorological factors) was carried out (Table 7 and Table 8). The fruit size (length, width of fruit, and fresh weight of fruit) showed a positive correlation with available phosphate (0.372, *p* < 0.01; 0.352, *p* < 0.01; 0.337, *p* < 0.01) and a negative correlation with exchangeable sodium (−0.310, *p* < 0.01; −0.254, *p* < 0.05; −0.255, *p* < 0.05), annual maximum temperature (−0.363, *p* < 0.01; −0.219, *p* < 0.05; −0.306, *p* < 0.01), and altitude (−0.325, *p* < 0.01; −0.412, *p* < 0.01; −0.245, *p* < 0.05). Additionally, the sugar contents of fruit showed a negative correlation with available phosphate (−0.309, *p* < 0.01), exchangeable potassium (−0.220, *p* < 0.05), and altitude (0.279, *p* < 0.05). Furthermore, the number of seeds was negatively correlated with the annual maximum temperature (−0.221, *p* < 0.05). 

Correlation analysis between the active compounds and growth characteristics was also carried out (Table 9). The fruit size (length, width of fruit, and fresh weight of fruit) showed a negative correlation with 6,8-diprenylorobol (−0.436, *p* < 0.01; −0.327, *p* < 0.01; −0.447, *p* < 0.01), 6,8-diprenylgenistein (−0.401, *p* < 0.01; −0.351, *p* < 0.01; −0.418, *p* < 0.01), and total contents (−0.326, *p* < 0.01; −0.267, *p* < 0.05; −0.301, *p* < 0.01). The sugar contents of fruit were positively correlated with 6,8-diprenylorobol (0.310, *p* < 0.01) and 6,8-diprenylgenistein (0.281, *p* < 0.01). Additionally, the aspect ratio was positively correlated with 6,8-diprenylorobol. However, 4′-*O*-methylalpinumisoflavone was not correlated with any growth characteristics.

## 3. Discussion

We collected fruits of *C. tricuspidata* at 28 cultivation sites in this study. The soil characteristics of the *C. tricuspidata* cultivation site were compared with the physicochemical properties (Table 1) suitable for plant growth [40]. The pH values of only six cultivation sites were within the appropriate range (pH 5.5–6.5). The electrical conductivity (<1.0) was found to be suitable for all cultivation sites. Organic matter (≥3.0) and total nitrogen (≥0.25) were at good levels at 12 and 18 sites, respectively. Available phosphate (≥100) was generally suitable, except for at five cultivation sites. As for exchangeable cations, Ca^2+^, Mg^2+^, and Na^+^, except for K^+^, were not suitable at most cultivation sites in the proper range. In the case of cation exchange capacity, 19 cultivation sites were within the appropriate range (12.0–20.0). Base saturation was in the range of 8.12–101.74%. Base saturation was positively correlated with soil pH (0.790, *p* < 0.01, Table S3) [41]. In general, when the soil pH is lowered, the concentration of exchangeable aluminum increases, which is known to be a toxic substance that inhibits root development and plant growth by interrupting the absorption and movement of nutrients [42,43,44]. Additionally, high soil organic matter content increases crop productivity by enhancing nitrogen absorption in plants through nitrogen mineralization [45,46]. Meteorological data were also investigated (Table S1). The annual mean temperature ranged from 10.0 °C to 15.6 °C, with the highest annual mean temperature at 21.1 °C and the lowest annual mean temperature at 4.6 °C. The amount of total precipitation was also confirmed to vary from 923.0 mm to 1800.1 mm. *C. tricuspidata* is known to grow mainly on the coast [47], but it is also cultivated in mountainous areas. Environmental factors, such as temperature, precipitation, soil acidity, organic matter, soil drainage, etc., are very complex, and these heterogeneities have a significant impact on plant growth and the contents of secondary metabolites [48,49]. Even though studies have been conducted on how the growth of plants is changed by environmental factors [50,51,52], there are not many studies on the effect on the properties of active compounds. The active compounds of plants are affected by a variety of factors, including weather, soil, and genetic factors, and each component is synthesized through different pathways in different environments [53].

The size of the *C. tricuspidata* fruits was similar to previously reported results [54]. Overall, the width of the fruit was longer than the length of the fruit, and the fresh weight and sugar contents of the fruit were found to be of various values. This difference in fruit growth characteristics at different cultivation sites may be owing to soil heterogeneity and meteorological factors. It was reported that soil properties, such as organic matter, soil acidity, and drainage, and meteorological factors, such as altitude, precipitation, and temperature, affect plant growth [55,56]. 

Analytical methods for simultaneous quantitation of major compositions in *C. tricuspidata* fruits were developed and validated in this study. Significant method validation results for linearity, LOD, LOQ, precision, and accuracy showed that the analytical method provided reproducible and reliable results. By using a validated analytical method, all major compositions were detected within 15 min (Figure 2). It was found that 6,8-diprenylgenistein was the most abundant in the present analytical method, and the same result was reported in previous studies [22,57]. By analyzing the growth characteristics of *C. tricuspidata* fruits and environmental factors, it was found that the size of fruits (length of fruit, width of fruit, and fresh weight of fruit) had a negative correlation with exchangeable sodium, annual maximum temperature, and altitude, and there was a positive correlation with available phosphate. In the previous studies, the electrical conductivity of non-saline soils was less than 2.0 dS/m in the standards of salinity soil, and the exchangeable sodium percentage was less than 15% [58,59]. The electrical conductivity of the soil collected in this study was 0.49 ds/m for the highest cultivation site and 2.77% for the highest exchangeable sodium percentage. It is judged that the exchangeable sodium, which showed a significant correlation, does not have a significant effect on the growth of *C. tricuspidata*. It has been reported that available phosphate is a plant cell constituent substance that is involved in the development of roots, leaves, flowers, and fruits and plays an important role in intermediate metabolites and energy metabolism in the growth of plants [60]. Zhang et al. (2018) maintained that the average, minimum, and maximum temperatures from April to October had the most positive effect on the quality of Fuji apple fruit, followed by annual average temperature and sunshine, the temperature difference between day and night, and the total precipitation during the same period [61]. Additionally, In the correlation between fruit growth characteristics and active compounds, the contents of 6,8-diprenylorobol and 6,8-diprenylgenistein and the total contents had a negative correlation with the size of the fruit. These results are similar to previous studies that found a negative correlation between plant growth and secondary metabolites. Park et al. (2020) reported that there was a negative correlation between the dry weight of roots and active compounds (nodakenin, decursin, and decursinol angelate) in *Angelica gigas* Nakai [27]. Additionally, Trujillo-Mayol et al. (2020) then reported that the weight and volume of fruit had a strong negative correlation between total flavonoid content and total phenyl content in avocado fruits, and enzymes such as phenylalanine ammonia-lyase, chalcone synthase, and polyphenol oxidase, involved in the biosynthesis of the polyphenol compounds, also have a negative correlation [62]. Additionally, Cunha et al. (2023) reported that selenium promoted leaf growth in *glycine max* L. and increased the content of carotenoids and the activity of antioxidant enzymes (superoxide dismutase, catalase, ascorbate peroxide) [63]. Wu et al. (2022) reported that the appearance of blueberry leaves differs according to various shading treatments, and the content of metabolites also appears differently [64]. These studies confirm that the environmental conditions with the highest flavonoid content may not be the same as the best growth conditions for plants. Therefore, it is possible to proceed with cultivation by changing the cultivation conditions for use as food or medicine.

## 4. Materials and Methods

### 4.1. Plant Materials and Chemicals

**Figure 3:**
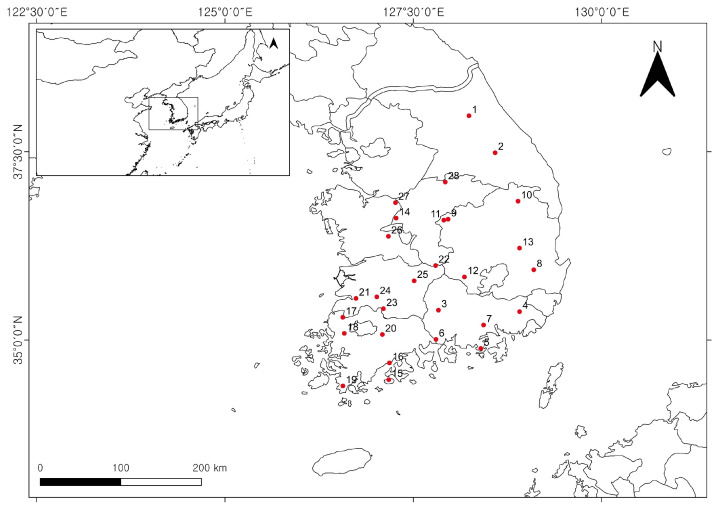
Geographical information for the cultivated samples of *Cudrania tricuspidata* from the various provinces.

A total of 133 specimens of *C. tricuspidata* fruits were collected from 28 cultivation sites across South Korea in October 2021 (Table S4 and Figure 3). Plant taxonomic identification was carried out for the samples, stored at 2 °C. Standards of 6,8-diprenylorobol (≥98%, CAS No. 66777-70-6) and 6,8-diprenylgenistein (≥98%, CAS No. 51225-28-6) were purchased from ChemFaces (Wuhan, China). 4′-*O*-methylalpinumisoflavone (≥95%, CAS No. 27762-87-4) was purchased from NIKOM (Gyeongsan, Republic of Korea). Methanol, acetonitrile, and distilled water were of HPLC grade and purchased from J.T. Baker (Avantor, Inc., Radnor, PA, USA).

### 4.2. Soil Analysis

Soils were collected at a depth within 20 cm after removing surface soils at cultivation sites. Collected soil samples were passed through a 2 mm sieve and dried at room temperature. Soils were classified according to United States Department of Agriculture (USDA) specification into 12 classes of soil texture classification. The soil physicochemical properties analyses, such as soil pH, electrical conductivity (EC), organic matter (OM), total nitrogen (TN), available phosphate (Avail. P_2_O_5_), exchangeable cation (K^+^, Ca^2+^, Mg^2+^, and Na^+^), and cation exchange capacity (CEC), were performed following the standard analysis manual of the Rural Development Administration (RDA) in Korea. Base saturation (BS) was calculated from Equation (1) as follows:

(1) $$\mathrm{Base}\;\mathrm{saturation}\;\left(\%\right)=\frac{\mathrm{Exchangeable}\;\mathrm{cation}}{\mathrm{Cation}\;\mathrm{exchange}\;\mathrm{capacity}}\;\times \;100$$

### 4.3. Meteorological Data

Data on weather conditions at the cultivation sites were collected using the Korea Meteorological Administration’s open portal (data.kma.go.kr). The collected data were annual mean temperature, annual mean maximum temperature, annual mean minimum temperature, annual maximum temperature, annual minimum temperature, and total precipitation.

### 4.4. Growth Characteristics

Fruits of *C. tricuspidate* showed moisture content from 17.68 ± 0.86% to 30.31 ± 0.77%. Quantitative characteristics of *C. tricuspidate*, such as length, width, and fresh weight of fruit; number of seeds; and sugar contents of fruit, were measured using digital calipers (500-182-30, Mitutoyo Co., Kawasaki, Japan), electronic scale (HS3200S, HANSUNG instrument Co., Gwangmyeong, Republic of Korea), and refractometer (PR-101α, ATAGO Co., Ltd., Tokyo, Japan). Aspect ratio of fruit was calculated from Equation (2) as follows:

(2) $$\mathrm{Aspect}\;\mathrm{ratio}=\frac{\mathrm{Width}\;\mathrm{of}\;\mathrm{fruit}}{\mathrm{Length}\;\mathrm{of}\;\mathrm{fruit}}$$

### 4.5. Sample and Standard Preparation

The collected samples were washed with distilled water and then lyophilized. After measuring the dry weight of the sample, samples were pulverized with a grinder (KSP-35, Korea Medi Co., Ltd., Daegu, Republic of Korea) and then passed through an 80 mesh standard sieve, stored at −18 °C, and used as an analysis sample.

Dried sample (1 g) was extracted with 20 mL of 100% methanol in an ultrasonic bath (JAC-5020, KODO, Hwaseong, Republic of Korea). The output power was 350 W, and the frequency was 40 kHz of the ultrasonic bath. The sonication temperature and time were 30 °C and 60 min. The extracts were centrifuged (Labogene UM-1248, Bio-Medical Science Co. Ltd., Seoul, Republic of Korea) for 10 min at 1763× *g*, and the supernatant was separated. The supernatant was filtered by a 0.2 µm membrane filter (Whatman Co., Maidstone, UK). Standard (6,8-diprenylorobol, 6,8-diprenylgenistein, and 4′-*O*-methylalpinumisoflavone) stock solutions for UPLC were prepared by diluting the stock solutions in methanol to obtain concentration ranges of 6.25–200 µg/mL for 6,8-diprenylorobol, 5–600 µg/mL for 6,8-diprenylgenistein, and 12.5–400 µg/mL for 4′-*O*-methylalpinumisoflavone.

### 4.6. UPLC Conditions

Waters alliance UPLC^®^ (Waters Co., Milford, MA, USA) with a UV detector was used for simultaneous quantification of major compound in *C. tricuspidata* fruit. The analytical conditions for three compounds were as follows: qualitative and quantitative analyses were carried out using an ACQUITY UPLC BEH C18 column (2.1 × 100 mm, 1.7 µm, 130 Å, Waters Co., Milford, MA, USA) with a column oven at 30 °C. The mobile phase was a binary eluent of 0.1% formic acid in water (A) and 0.1% formic acid in acetonitrile (B) with gradient conditions as follows: initial–2 min, 40% B; 2–18 min, 80% B; 18–18.1 min, 100% B; 18.1–20 min, 100% B; injection volume of 1 µL, flow rate of 0.3 mL/min, and detection wavelength of 260 nm. Each sample was analyzed in triplicate and expressed as a mean value.

### 4.7. Method Validation

The UPLC-UV method was validated by testing linearity, limits of detection (LOD), limits of quantification (LOQ), precision, and accuracy. Calibration curves were constructed with six different concentrations from the following concentration ranges: 6.25–200 µg/mL for 6,8-diprenylorobol; 5–600 µg/mL for 6,8-diprenylgenistein; 12.5–400 µg/mL for 4′-*O*-methylalpinumisoflavone. LOD and LOQ were calculated from Equations (3) and (4) as follows:

(3) $$\mathrm{LOD}\;(µg/\mathrm{mL})=3.3\;\times \frac{\sigma }{S}$$

(4) $$\mathrm{LOD}\;(µg/\mathrm{mL})=10\;\times \frac{\sigma }{S}$$

where *σ* and *S* are the standard deviation (SD) of the *y*-intercept and the slope of the calibration curve, respectively.

The precision (%) was evaluated with three different concentrations in triplicates within one day (intra-day) or three sequential days (inter-day). The intra- and inter-day precisions were expressed as the relative standard deviation (RSD). The RSD was calculated by Equation (5) as follows:

(5) $$\mathrm{RSD}\;\left(\%\right)=\frac{\mathrm{SD}}{\mathrm{Mean}}\;\times \;100$$

Accuracy (%) was evaluated using a recovery assay which was examined by analyzing the peak areas of standard stock solution spiked sample extracts and peak areas of sample extracts with no stock solution added. Recovery was calculated by Equation (6) as follows:

(6) $$\mathrm{Recovery}\;\left(\%\right)=\frac{\mathrm{Measured}\;\mathrm{amount}}{\mathrm{Spiked}\;\mathrm{amount}}\;\times \;100$$

Each sample was analyzed in triplicate at three different concentrations and expressed as a mean value. 

### 4.8. Statistical and Multivariate Analysis

Statistical analysis was performed using SPSS software (Statistical Package for the Social Science, Version 26, IBM SPSS statistics, Chicago, IL, USA), and data were expressed as mean ± standard error (S.E.). Statistical analyses of the results were performed at a 5% significance level. One-way analysis of variance (ANOVA) and Duncan’s multiple-range test were applied to address the differences. The correlation among growth characteristics, active compounds of *C. tricuspidata* fruit, and the environment of the cultivation site was confirmed by Pearson’s correlation coefficient.

## 5. Conclusions

We developed a method for the simultaneous analysis of major compounds of *C. tricuspidata* fruit based on the UPLC-UV. The method was validated with respect to linearity, LOD, LOQ, precision, and accuracy. The growth characteristics and active compounds contents of *C. tricuspidata* fruit at 28 cultivation regions across South Korea were investigated in this study. The correlation among active compounds, growth characteristics, and the environment was also analyzed. 6,8-diprenylorobol, 6,8-diprenylgenistein, and total contents had a negative correlation with the fruit size, and the fruit size showed a positive correlation with available phosphate and a negative correlation with exchangeable sodium, annual maximum temperature, and altitude. These results can be used to study the standard cultural practices and quality control of *C. tricuspidata* fruits.

## Figures and Tables

**Table 1:** Soil physicochemical properties of 28 different *Cudrania tricuspidata* cultivation sites.

Cultivation Sites (*n* = 3)	Soil Texture	pH [1:5]	EC [1:5]	OM	TN	Avail. P_2_O_5_	Exchangeable Cation				CEC	BS
							K^+^	Ca^2+^	Mg^2+^	Na^+^		
			(dS/m)	(%)	(%)	(mg/kg)	(cmol^+^/kg)				(cmol^+^/kg)	(%)
1	sandy loam	4.24 ± 0.04 ^j^	0.19 ± 0.03 ^efgh^	3.88 ± 0.38 ^cdefgh^	0.25 ± 0.03 ^cdefgh^	53.07 ± 17.10 ^gh^	0.29 ± 0.02 ^fgh^	1.20 ± 0.12 ^ij^	0.25 ± 0.03 ^hij^	0.29 ± 0.00 ^abcde^	16.69 ± 0.30 ^cdef^	12.14 ± 1.13 ^j^
2	loam	4.96 ± 0.12 ^efghij^	0.14 ± 0.02 ^fgh^	2.37 ± 0.21 ^fghij^	0.13 ± 0.00 ^gh^	38.72 ± 07.62 ^h^	0.23 ± 0.04 ^h^	5.81 ± 0.36 ^cdefghij^	0.55 ± 0.03 ^ghij^	0.13 ± 0.02 ^def^	13.27 ± 0.18 ^fg^	50.59 ± 0.50 ^defgh^
3	sandy loam	4.28 ± 0.09 ^j^	0.17 ± 0.01 ^fgh^	3.47 ± 0.21 ^cdefghij^	0.20 ± 0.00 ^defgh^	49.11 ± 05.87 ^gh^	0.38 ± 0.08 ^defgh^	1.41 ± 0.16 ^hij^	0.43 ± 0.10 ^hij^	0.20 ± 0.03 ^cdef^	18.58 ± 0.16 ^bcde^	12.99 ± 0.63 ^j^
4	sandy loam	6.04 ± 0.20 ^bc^	0.23 ± 0.04 ^defgh^	3.65 ± 0.88 ^cdefghi^	0.23 ± 0.04 ^cdefgh^	103.35 ± 55.89 ^gh^	0.48 ± 0.03 ^cdefgh^	13.05 ± 1.77 ^ab^	1.84 ± 0.29 ^bcdef^	0.08 ± 0.00 ^f^	19.31 ± 1.59 ^bcd^	79.44 ± 0.21 ^abc^
5	sandy loam	5.45 ± 0.18 ^cde^	0.35 ± 0.01 ^bcd^	5.16 ± 0.22 ^cd^	0.31 ± 0.02 ^cd^	43.87 ± 03.84 ^gh^	0.53 ± 0.10 ^cdefgh^	9.99 ± 0.87 ^bcde^	1.06 ± 0.09 ^cdefghij^	0.12 ± 0.01 ^def^	20.63 ± 0.10 ^bcd^	56.66 ± 0.21 ^cdef^
6	sandy loam	4.38 ± 0.10 ^hij^	0.14 ± 0.01 ^fgh^	3.34 ± 0.55 ^defghij^	0.20 ± 0.04 ^defgh^	199.32 ± 43.58 ^fgh^	0.23 ± 0.02 ^h^	0.67 ± 0.17 ^j^	0.11 ± 0.01 ^j^	0.35 ± 0.04 ^abc^	16.84 ± 0.41 ^cdef^	8.12 ± 1.42 ^j^
7	sandy loam	5.31 ± 0.19 ^cde^	0.24 ± 0.02 ^cdefgh^	5.92 ± 0.21 ^bc^	0.32 ± 0.00 ^bcd^	238.69 ± 46.56 ^fgh^	0.75 ± 0.31 ^abcd^	10.67 ± 4.24 ^bcd^	1.35 ± 0.19 ^bcdefghi^	0.34 ± 0.07 ^abc^	20.40 ± 2.25 ^bcd^	60.89 ± 0.06 ^cde^
8	sandy loam	5.16 ± 0.49 ^defg^	0.23 ± 0.07 ^defgh^	4.75 ± 1.37 ^cdef^	0.27 ± 0.06 ^cdefg^	115.98 ± 31.88 ^gh^	0.47 ± 0.16 ^cdefgh^	11.44 ± 3.12 ^abc^	2.29 ± 0.74 ^ab^	0.42 ± 0.20 ^ab^	21.12 ± 2.68 ^bc^	67.46 ± 0.09 ^cde^
9	sandy loam	5.57 ± 0.39 ^cde^	0.36 ± 0.06 ^bcd^	7.65 ± 0.95 ^ab^	0.43 ± 0.04 ^ab^	591.22 ± 72.42 ^cde^	1.12 ± 0.26 ^a^	12.92 ± 4.22 ^ab^	2.19 ± 0.72 ^abc^	0.44 ± 0.10 ^a^	26.40 ± 2.67 ^a^	60.19 ± 0.07 ^cde^
10	sandy loam	5.40 ± 0.20 ^cde^	0.13 ± 0.01 ^gh^	1.49 ± 0.21 ^hij^	0.12 ± 0.00 ^h^	53.83 ± 06.51 ^gh^	0.17 ± 0.00 ^h^	5.79 ± 0.62 ^cdefghij^	0.63 ± 0.18 ^ghij^	0.30 ± 0.10 ^abcd^	10.96 ± 0.88 ^g^	62.54 ± 0.37 ^cde^
11	sandy loam	6.75 ± 0.34 ^a^	0.24 ± 0.02 ^cdefgh^	3.07 ± 0.49 ^defghij^	0.20 ± 0.03 ^defgh^	180.35 ± 38.25 ^fgh^	0.28 ± 0.06 ^fgh^	16.84 ± 2.81 ^a^	0.84 ± 0.36 ^fghij^	0.35 ± 0.14 ^abc^	19.67 ± 1.19 ^bcd^	92.36 ± 0.11 ^ab^
12	sandy loam	5.08 ± 0.19 ^defghi^	0.24 ± 0.01 ^cdefgh^	3.47 ± 0.14 ^cdefghij^	0.21 ± 0.01 ^defgh^	188.11 ± 49.41 ^fgh^	0.34 ± 0.08 ^defgh^	11.94 ± 3.05 ^abc^	1.01 ± 0.11 ^cdefghij^	0.20 ± 0.01 ^cdef^	17.95 ± 0.70 ^bcdef^	74.30 ± 0.07 ^bcd^
13	sandy loam	5.75 ± 0.12 ^bcd^	0.18 ± 0.02 ^efgh^	2.56 ± 0.48 ^efghij^	0.16 ± 0.02 ^efgh^	182.56 ± 49.77 ^fgh^	0.33 ± 0.01 ^efgh^	7.98 ± 0.64 ^bcdefg^	1.73 ± 0.05 ^bcdefg^	0.23 ± 0.02 ^bcdef^	16.98 ± 0.17 ^cdef^	60.49 ± 0.30 ^cde^
14	sandy loam	5.63 ± 0.12 ^bcde^	0.15 ± 0.01 ^fgh^	1.06 ± 0.10 ^j^	0.13 ± 0.00 ^gh^	793.83 ± 126.72 ^bcd^	0.30 ± 0.03 ^fgh^	7.16 ± 0.42 ^bcdefghi^	3.18 ± 0.29 ^a^	0.10 ± 0.01 ^ef^	10.58 ± 0.16 ^g^	101.74 ± 0.20 ^a^
15	sandy loam	4.54 ± 0.17 ^fghij^	0.28 ± 0.04 ^bcdef^	5.01 ± 0.68 ^cde^	0.30 ± 0.03 ^cde^	789.79 ± 155.93 ^bcd^	0.66 ± 0.21 ^cdefg^	2.52 ± 0.80 ^ghij^	1.24 ± 0.57 ^bcdefghij^	0.11 ± 0.02 ^def^	18.16 ± 0.36 ^bcdef^	24.76 ± 0.12 ^ij^
16	sandy loam	4.36 ± 0.25 ^ij^	0.27 ± 0.01 ^bcdefg^	2.94 ± 0.08 ^defghij^	0.20 ± 0.01 ^defgh^	933.97 ± 51.90 ^ab^	0.80 ± 0.19 ^abc^	3.78 ± 0.56 ^efghij^	0.98 ± 0.10 ^defghij^	0.08 ± 0.03 ^f^	16.71 ± 0.34 ^cdef^	33.85 ± 0.25 ^fghij^
17	sandy loam	6.32 ± 0.10 ^ab^	0.38 ± 0.04 ^abc^	4.35 ± 0.52 ^cdefg^	0.28 ± 0.04 ^cdef^	858.55 ± 141.55 ^abc^	0.88 ± 0.18 ^abc^	10.74 ± 1.86 ^bcd^	1.43 ± 0.29 ^bcdefgh^	0.10 ± 0.02 ^ef^	19.81 ± 1.09 ^bcd^	65.41 ± 0.12 ^cde^
18	sandy loam	5.23 ± 0.33 ^defg^	0.31 ± 0.04 ^bcde^	4.95 ± 0.57 ^cde^	0.36 ± 0.05 ^bc^	441.65 ± 83.63 ^ef^	0.68 ± 0.14 ^bcdef^	7.62 ± 1.73 ^bcdefgh^	2.12 ± 0.79 ^abcd^	0.06 ± 0.00 ^f^	22.73 ± 1.84 ^ab^	44.99 ± 0.13 ^efghi^
19	sandy loam	4.50 ± 0.08 ^ghij^	0.11 ± 0.01 ^h^	1.97 ± 0.14 ^ghij^	0.15 ± 0.01 ^fgh^	537.79 ± 122.07 ^de^	0.21 ± 0.03 ^h^	0.76 ± 0.22 ^j^	0.16 ± 0.03 ^ij^	0.06 ± 0.01 ^f^	14.20 ± 0.95 ^efg^	8.31 ± 0.62 ^j^
20	sandy loam	4.21 ± 0.05 ^j^	0.20 ± 0.00 ^efgh^	4.36 ± 0.89 ^cdefg^	0.27 ± 0.04 ^cdefg^	158.81 ± 21.94 ^fgh^	0.19 ± 0.02 ^h^	2.74 ± 1.47 ^ghij^	0.61 ± 0.39 ^ghij^	0.09 ± 0.03 ^ef^	15.97 ± 0.73 ^def^	23.88 ± 0.07 ^ij^
21	sandy loam	5.28 ± 0.58 ^def^	0.25 ± 0.01 ^cdefgh^	2.77 ± 0.23 ^defghij^	0.21 ± 0.01 ^defgh^	796.16 ± 161.84 ^bcd^	0.71 ± 0.11 ^bcde^	6.63 ± 1.56 ^cdefghij^	2.11 ± 0.74 ^abcd^	0.09 ± 0.02 ^ef^	17.13 ± 0.51 ^cdef^	55.22 ± 0.08 ^cdefg^
22	loam	4.42 ± 0.19 ^hij^	0.24 ± 0.03 ^cdefgh^	3.75 ± 0.83 ^cdefgh^	0.26 ± 0.04 ^cdefgh^	878.36 ± 168.52 ^abc^	0.25 ± 0.02 ^gh^	3.90 ± 1.28 ^efghij^	0.91 ± 0.23 ^efghij^	0.04 ± 0.00 ^f^	16.99 ± 1.01 ^cdef^	29.14 ± 0.13 ^hij^
23	sandy loam	5.29 ± 0.17 ^def^	0.16 ± 0.03 ^fgh^	1.96 ± 0.47 ^ghij^	0.16 ± 0.03 ^efgh^	1105.91 ± 118.28 ^a^	0.37 ± 0.13 ^defgh^	4.50 ± 0.66 ^defghij^	0.77 ± 0.15 ^fghij^	0.13 ± 0.01 ^def^	14.06 ± 2.03 ^efg^	41.36 ± 0.27 ^efghi^
24	sandy loam	5.11 ± 0.09 ^defgh^	0.49 ± 0.16 ^a^	8.13 ± 2.56 ^a^	0.49 ± 0.15 ^a^	882.20 ± 227.9^4 abc^	0.69 ± 0.10 ^bcdef^	11.29 ± 3.32 ^abc^	2.06 ± 0.35 ^bcde^	0.07 ± 0.01 ^f^	26.55 ± 4.42 ^a^	51.99 ± 0.15 ^defgh^
25	sandy loam	4.56 ± 0.13 ^fghij^	0.25 ± 0.02 ^cdefgh^	2.79 ± 0.17 ^defghij^	0.19 ± 0.00 ^defgh^	184.18 ± 37.68 ^fgh^	0.25 ± 0.01 ^gh^	6.83 ± 0.38 ^bcdefghij^	1.40 ± 0.09 ^bcdefgh^	0.12 ± 0.03 ^def^	16.94 ± 0.33 ^cdefg^	50.77 ± 0.46 ^defgh^
26	sandy loam	5.74 ± 0.09 ^bcd^	0.40 ± 0.06 ^ab^	4.27 ± 0.89 ^cdefg^	0.28 ± 0.04 ^cdef^	356.51 ± 29.06 ^efg^	0.82 ± 0.05 ^abc^	9.05 ± 1.62 ^bcdef^	1.12 ± 0.10 ^bcdefghij^	0.06 ± 0.01 ^f^	19.50 ± 1.22 ^bcd^	56.03 ± 0.18 ^cdefg^
27	sandy loam	5.17 ± 0.08 ^defg^	0.25 ± 0.03 ^cdefgh^	2.67 ± 0.31 ^defghij^	0.20 ± 0.01 ^defgh^	622.02 ± 93.29 ^cde^	1.07 ± 0.14 ^ab^	3.57 ± 0.33 ^fghij^	0.58 ± 0.05 ^ghij^	0.11 ± 0.04 ^def^	17.56 ± 0.61 ^cdef^	30.42 ± 1.07 ^ghij^
28	sandy loam	5.26 ± 0.02 ^def^	0.17 ± 0.02 ^fgh^	1.14 ± 0.36 ^ij^	0.12 ± 0.00 ^h^	122.60 ± 29.29 ^gh^	0.15 ± 0.05 ^h^	4.96 ± 0.43 ^defghij^	0.60 ± 0.11 ^ghij^	0.12 ± 0.03 ^def^	10.34 ± 1.61 ^g^	58.25 ± 0.14 ^cdef^

EC: Electrical Conductivity; OM: Organic Matter; TN: Total Nitrogen; Avail. P_2_O_5_: Available Phosphate; CEC: Cation Exchange Capacity; BS: Base Saturation. Different letters in columns are significantly different using one-way ANOVA and Duncan’s multiple range test (*p* < 0.05).

**Table 2:** Growth characteristics of *Cudrania tricuspidata* fruit at 28 different cultivation sites.

Cultivation Sites (*n* = 3)	Length of Fruit	Width of Fruit	Fresh Weight of Fruit	Sugar Contents of Fruit	Number of Seeds	Aspect Ratio
	(mm)	(mm)	(g)	(Brix°)		
1	19.53 ± 0.37 ^m^	23.66 ± 0.67 ^k^	7.64 ± 0.76 ^j^	13.71 ± 2.06 ^bcdefgh^	9.78 ± 1.87 ^k^	1.21 ± 0.01 ^abcd^
2	28.75 ± 0.30 ^bcdef^	32.46 ± 0.99 ^bcdef^	20.41 ± 1.08 ^abc^	12.73 ± 1.18 ^cdefghij^	31.89 ± 2.80 ^a^	1.13 ± 0.02 ^cde^
3	25.72 ± 0.83 ^fghij^	29.07 ± 0.72 ^efghij^	14.17 ± 0.75 ^fghi^	13.38 ± 0.41 ^bcdefghi^	24.67 ± 2.17 ^abcdef^	1.13 ± 0.01 ^cde^
4	26.42 ± 0.94 ^fgh^	33.81 ± 0.55 ^bcd^	14.35 ± 0.57 ^efghi^	15.14 ± 0.67 ^bcdef^	17.11 ± 2.70 ^efghijk^	1.29 ± 0.07 ^a^
5	26.25 ± 1.77 ^fghi^	29.90 ± 1.94 ^defghi^	13.67 ± 2.31 ^fghi^	11.54 ± 1.76 ^fghij^	20.89 ± 4.57 ^bcdefghi^	1.14 ± 0.02 ^bcde^
6	19.89 ± 0.87 ^lm^	24.94 ± 0.56 ^jk^	7.53 ± 0.72 ^j^	19.40 ± 1.10 ^a^	9.33 ± 0.51 ^k^	1.26 ± 0.03 ^ab^
7	22.77 ± 0.23 ^jkl^	25.70 ± 1.06 ^ijk^	10.06 ± 0.97 ^ij^	12.57 ± 1.08 ^cdefghij^	21.33 ± 5.36 ^bcdefgh^	1.14 ± 0.05 ^bcde^
8	28.02 ± 1.31 ^cdefg^	33.36 ± 3.51 ^bcde^	16.59 ± 2.99 ^cdefgh^	10.86 ± 0.21 ^hij^	26.44 ± 6.22 ^abcde^	1.19 ± 0.07 ^abcde^
9	26.42 ± 1.78 ^fgh^	29.79 ± 1.77 ^defghi^	13.57 ± 2.82 ^fghi^	12.04 ± 2.24 ^defghij^	30.11 ± 2.84 ^ab^	1.13 ± 0.03 ^cde^
10	29.87 ± 1.31 ^bcde^	32.76 ± 0.69 ^bcde^	20.05 ± 2.34 ^abcd^	12.42 ± 0.50 ^cdefghij^	29.11 ± 3.93 ^abc^	1.10 ± 0.02 ^de^
11	25.64 ± 0.19 ^fghijk^	28.06 ± 0.21 ^fghij^	11.20 ± 0.93 ^hij^	14.96 ± 0.38 ^bcdefg^	11.78 ± 2.47 ^hijk^	1.10 ± 0.01 ^de^
12	24.91 ± 0.23 ^ghijk^	29.7 ± 1.46 ^efghij^	13.24 ± 0.93 ^fghi^	12.54 ± 0.61 ^cdefghij^	16.33 ± 1.02 ^fghijk^	1.17 ± 0.06 ^abcde^
13	23.03 ± 1.52 ^ijk^	26.41 ± 0.59 ^hijk^	13.17 ± 1.39 ^fghi^	15.71 ± 0.36 ^bcd^	11.22 ± 2.21 ^ijk^	1.15 ± 0.06 ^bcde^
14	28.67 ± 0.96 ^bcdef^	31.31 ± 2.56 ^bcdefg^	15.32 ± 0.88 ^cdefghi^	11.72 ± 0.90 ^efghij^	27.67 ± 1.02 ^abcd^	1.10 ± 0.05 ^de^
15	22.41 ± 1.05 ^klm^	27.73 ± 1.27 ^ghijk^	11.00 ± 0.55 ^hij^	12.34 ± 0.82 ^cdefghij^	10.33 ± 1.53 ^jk^	1.25 ± 0.04 ^abc^
16	30.81 ± 0.89 ^abc^	35.47 ± 1.48 ^ab^	23.76 ± 2.25 ^a^	10.86 ± 0.97 ^hij^	26.67 ± 3.50 ^abcde^	1.15 ± 0.02 ^bcde^
17	28.48 ± 1.47 ^cdef^	32.54 ± 0.85 ^bcdef^	17.03 ± 1.05 ^bcdefg^	11.19 ± 1.19 ^hij^	28.78 ± 3.09 ^abc^	1.15 ± 0.04 ^bcde^
18	30.56 ± 0.88 ^abc^	35.04 ± 0.31 ^abc^	19.89 ± 0.94 ^abcde^	9.47 ± 0.33 ^j^	19.89 ± 0.29 ^cdefghij^	1.15 ± 0.04 ^bcde^
19	26.80 ± 0.70 ^efgh^	31.06 ± 0.83 ^bcdefg^	15.37 ± 0.74 ^cdefghi^	11.38 ± 0.71 ^ghij^	16.67 ± 1.02 ^fghijk^	1.16 ± 0.04 ^abcde^
20	30.31 ± 0.66 ^abcd^	33.44 ± 0.55 ^bcde^	22.16 ± 1.52 ^ab^	13.14 ± 1.21 ^bcdefghij^	32.67 ± 3.10 ^a^	1.11 ± 0.01 ^de^
21	33.25 ± 1.53 ^a^	38.94 ± 2.84 ^a^	24.58 ± 4.49 ^a^	10.98 ± 1.07 ^hij^	18.78 ± 2.82 ^defghijk^	1.17 ± 0.03 ^abcde^
22	29.08 ± 0.90 ^bcdef^	31.93 ± 0.24 ^bcdefg^	17.14 ± 1.33 ^bcdefg^	13.46 ± 2.11 ^bcdefghi^	17.22 ± 1.79 ^efghijk^	1.11 ± 0.04 ^de^
23	31.90 ± 0.18 ^ab^	35.46 ± 0.19 ^ab^	25.29 ± 1.47 ^a^	9.78 ± 1.29 ^ij^	23.89 ± 2.16 ^abcdef^	1.11 ± 0.00 ^de^
24	26.65 ± 0.62 ^efgh^	30.64 ± 0.41 ^cdefgh^	13.67 ± 0.83 ^fghi^	16.42 ± 0.61 ^ab^	18.33 ± 0.96 ^defghijk^	1.15 ± 0.02 ^bcde^
25	28.39 ± 1.00 ^cdef^	31.67 ± 1.61 ^bcdefg^	16.67 ± 1.58 ^bcdefgh^	15.43 ± 0.64 ^bcde^	23.11 ± 2.33 ^abcdefg^	1.12 ± 0.04 ^de^
26	27.14 ± 0.76 ^defgh^	31.24 ± 0.85 ^bcdefg^	14.69 ± 0.20 ^defghu^	15.94 ± 1.18 ^ab^	26.00 ± 3.93 ^abcdef^	1.16 ± 0.05 ^bcde^
27	31.23 ± 0.76 ^abc^	33.57 ± 0.67 ^bcde^	18.09 ± 1.72 ^bcdef^	10.52 ± 0.23 ^hij^	19.89 ± 2.26 ^cdefghij^	1.08 ± 0.01 ^e^
28	23.78 ± 1.17 ^hijk^	27.74 ± 0.99 ^ghijk^	11.98 ± 1.35 ^ghij^	15.40 ± 0.21 ^bcde^	14.00 ± 2.01 ^ghijk^	1.18 ± 0.03 ^abcde^

Different letters in columns are significantly different using one-way ANOVA and Duncan’s multiple range test (*p* < 0.05).

**Table 3:** Linear regression, LOD, and LOQ of three active compounds.

Compound	Regression Equation	Correlation Coefficient (*r*^2^)	Range (µg/mL)	LOD (µg/mL)	LOQ (µg/mL)
6,8-diprenylorobol	Y = 15,750X − 32,632	0.9997	6.25–200	0.01	0.05
6,8-diprenylgenistein	Y = 14,552X − 70,083	0.9996	5–600	0.03	0.09
4′-*O*-methylalpinumisoflavone	Y = 6514.5X + 36,219	0.9992	12.5–400	0.01	0.04

**Table 4:** Intra- and inter-day precision of three active compounds.

Compound	Concentration (µg/mL)	Intra-Day ^a^ (*n* = 3)		Inter-Day ^b^ (*n* = 3)	
		Concentration Found (µg/mL)	RSD (%)	Concentration Found (µg/mL)	RSD (%)
6,8-diprenylorobol	12.5	13.1	0.14	12.8	0.86
	50	53.6	0.56	50.5	1.05
	200	219.9	0.44	202.3	0.20
6,8-diprenylgenistein	100	102.5	0.14	101.0	0.27
	200	212.0	0.01	206.2	1.82
	400	432.6	0.29	416.0	1.02
4′-*O*-methylalpinumisoflavone	25	25.9	0.06	24.2	0.30
	100	102.9	0.12	103.9	0.75
	400	423.8	0.04	412.9	0.81

^a^ The sample was analyzed three times in one day, *n* = 3. ^b^ The sample was analyzed each day for three consecutive days, *n* = 3.

**Table 5:** Recoveries of three active compounds.

Compound	Concentration (µg/mL)	Recovery (%) (*n* = 3)	RSD (%)
6,8-diprenylorobol	6.25	101.19	0.61
	25	100.81	0.80
	100	98.18	1.05
6,8-diprenylgenistein	50	104.24	0.12
	200	100.52	0.67
	800	97.25	0.72
4′-*O*-methylalpinumisoflavone	25	98.29	0.45
	100	104.98	1.18
	400	101.62	0.28

**Table 6:** Active compounds composition of *Cudrania tricuspidata* at 28 different cultivation sites.

Cultivation Sites (*n* = 3)	6,8-Diprenylorobol (µg/g)	6,8-Diprenylgenistein (µg/g)	4′-*O*-Methylalpinumisoflavone (µg/g)	Total (µg/g)
1	137.55 ± 47.73 ^a^	262.24 ± 24.40 ^ab^	245.89 ± 17.23 ^bc^	647.14 ± 61.06 ^a^
2	34.37 ± 08.98 ^efgh^	217.80 ± 39.03 ^bc^	108.73 ± 31.75 ^hijklmno^	362.15 ± 68.70 ^cdefg^
3	21.39 ± 00.69 ^fgh^	154.27 ± 07.77 ^cdef^	142.97 ± 14.30 ^fghijk^	322.73 ± 22.56 ^defgh^
4	76.43 ± 23.73 ^bcd^	157.35 ± 08.63 ^cdef^	115.75 ± 03.60 ^ghijklmn^	350.78 ± 25.78 ^cdefg^
5	91.31 ± 17.04 ^bc^	209.18 ± 03.17 ^bcd^	171.52 ± 76.76 ^defghi^	472.84 ± 93.57 ^bc^
6	47.07 ± 09.85 ^defgh^	213.05 ± 19.74 ^bcd^	179.75 ± 18.00 ^cdefgh^	441.64 ± 46.50 ^cd^
7	46.35 ± 02.09 ^defgh^	36.38 ± 01.60 ^ijk^	45.95 ± 00.68 ^nopqr^	129.16 ± 03.04 ^ijk^
8	73.93 ± 11.24 ^bcde^	114.32 ± 18.91 ^fghi^	129.16 ± 20.34 ^ghijklm^	318.26 ± 35.03 ^defgh^
9	103.37 ± 08.89 ^ab^	164.48 ± 22.84 ^cdef^	182.87 ± 18.62 ^cdefg^	452.44 ± 32.95 ^cd^
10	24.98 ± 02.07 ^fgh^	27.50 ± 02.40 ^jk^	31.46 ± 03.50 ^pqr^	88.01 ± 07.85 ^jk^
11	55.91 ± 02.34 ^cdef^	294.68 ± 17.97 ^a^	226.73 ± 07.51 ^bcde^	582.12 ± 27.77 ^ab^
12	23.15 ± 00.86 ^fgh^	65.73 ± 11.08 ^ghijk^	18.29 ± 02.80 ^qr^	108.70 ± 13.84 ^jk^
13	29.86 ± 00.50 ^fgh^	163.09 ± 05.59 ^cdef^	157.72 ± 04.35 ^efghij^	353.19 ± 09.70 ^cdefg^
14	33.38 ± 00.40 ^fgh^	198.41 ± 07.09 ^bcde^	202.48 ± 03.93 ^cdef^	437.52 ± 03.68 ^cd^
15	48.26 ± 00.97 ^defg^	166.49 ± 44.50 ^cdef^	98.52 ± 42.06 ^ijklmnop^	315.31 ± 56.27 ^defgh^
16	19.66 ± 02.43 ^fgh^	33.12 ± 01.56 ^ijk^	322.60 ± 11.90 ^a^	377.19 ± 09.74 ^cdef^
17	53.28 ± 15.79 ^cdefg^	231.96 ± 83.15 ^abc^	121.05 ± 18.70 ^ghijklm^	408.19 ± 109.04 ^cde^
18	17.82 ± 00.81 ^fgh^	21.13 ± 02.88 ^k^	288.34 ± 33.81 ^ab^	328.58 ± 37.35 ^defgh^
19	17.37 ± 01.86 ^fgh^	46.10 ± 05.98 ^hijk^	12.17 ± 02.38 ^r^	79.21 ± 10.17 ^jk^
20	13.39 ± 00.64 ^gh^	53.03 ± 20.60 ^ghijk^	139.11 ± 15.43 ^fghijkl^	207.72 ± 16.82 ^hij^
21	54.51 ± 01.77 ^cdefg^	122.23 ± 53.67 ^efgh^	115.63 ± 25.54 ^ghijklmn^	293.20 ± 60.09 ^efgh^
22	27.61 ± 03.64 ^fgh^	34.22 ± 05.29 ^ijk^	39.39 ± 05.92 ^opqr^	103.30 ± 14.78 ^jk^
23	16.00 ± 00.72 ^fgh^	19.89 ± 00.78 ^k^	234.27 ± 18.52 ^bcd^	271.37 ± 19.82 ^efgh^
24	90.53 ± 02.77 ^bc^	132.69 ± 03.78 ^defg^	70.37 ± 01.21 ^klmnopq^	294.61 ± 05.66 ^efgh^
25	35.13 ± 04.85 ^efgh^	111.78 ± 22.76 ^fghi^	85.91 ± 14.18 ^jklmnopq^	233.86 ± 27.83 ^ghi^
26	89.71 ± 06.85 ^bc^	104.16 ± 12.23 ^fghij^	59.28 ± 03.67 ^mnopqr^	254.97 ± 21.04 ^fghi^
27	6.37 ± 00.63 ^h^	27.77 ± 02.66 ^jk^	10.61 ± 02.14 ^r^	46.79 ± 04.96 ^k^
28	97.45 ± 07.05 ^b^	113.37 ± 04.29 ^fghi^	66.44 ± 00.90 ^lmnopqr^	281.02 ± 11.34 ^efgh^

Different letters in columns are significantly different using one-way ANOVA and Duncan’s multiple range test (*p* < 0.05).

**Table 7:** Pearson’s correlation coefficient between growth characteristics and soil physicochemical properties of *Cudrania tricuspidata*.

	Correlation Coefficient (*r*) ^a^					
	Length of Fruit	Width of Fruit	Fresh Weight of Fruit	Sugar Contents of Fruit	Number of Seeds	Aspect Ratio
pH	0.075 (0.500)	0.080 (0.472)	−0.053 (0.634)	−0.044 (0.691)	0.056 (0.615)	−0.034 (0.759)
EC	0.066 (0.550)	0.098 (0.378)	−0.031 (0.778)	0.033 (0.764)	0.089 (0.420)	0.023 (0.836)
OM	−0.155 (0.158)	−0.098 (0.373)	−0.213 (0.052)	0.064 (0.566)	0.032 (0.770)	0.114 (0.302)
TN	−0.080 (0.472)	−0.029 (0.794)	−0.146 (0.186)	0.042 (0.703)	0.033 (0.766)	0.093 (0.398)
Avail. P_2_O_5_	0.372 ** (0.000)	0.352 ** (0.001)	0.337 ** (0.002)	−0.309 ** (0.004)	0.055 (0.620)	−0.093 (0.401)
K^+^	0.120 (0.277)	0.117 (0.290)	−0.006 (0.959)	−0.220 * (0.044)	0.102 (0.354)	−0.047 (0.669)
Ca^2+^	−0.003 (0.976)	0.016 (0.882)	−0.128 (0.247)	0.032 (0.775)	0.046 (0.675)	−0.015 (0.890)
Mg^2+^	0.176 (0.109)	0.201 (0.066)	0.060 (0.588)	−0.191 (0.082)	0.188 (0.086)	−0.007 (0.950)
Na^+^	−0.310 ** (0.004)	−0.254 * (0.020)	−0.255 * (0.019)	0.082 (0.458)	−0.106 (0.336)	0.154 (0.162)
CEC	−0.095 (0.391)	−0.033 (0.768)	−0.191 (0.082)	0.022 (0.844)	−0.037 (0.739)	0.104 (0.346)
BS	0.259 * (0.018)	0.159 (0.152)	0.321 ** (0.003)	−0.036 (0.745)	0.455 ** (0.000)	−0.230 * (0.036)

^a^ Correlation coefficient (*r*) written is significantly correlated between the variables compared. Positive values denote positive correlation, and negative values denote negative correlation. Values in brackets indicate *p* value (** *p* < 0.01, * *p* < 0.05).

**Table 8:** Pearson’s correlation coefficient between growth characteristics and meteorological properties of *Cudrania tricuspidata*.

	Correlation Coefficient (*r*) ^a^					
	Length of Fruit	Width of Fruit	Fresh Weight of Fruit	Sugar Contents of Fruit	Number of Seeds	Aspect Ratio
Annual mean temp.	0.034 (0.757)	0.176 (0.110)	0.015 (0.889)	−0.089 (0.419)	−0.076 (0.491)	0.278 * (0.010)
Annual mean max temp.	−0.067 (0.546)	0.073 (0.508)	−0.031 (0.776)	−0.006 (0.959)	−0.127 (0.250)	0.293 ** (0.007)
Annual mean min temp.	0.069 (0.535)	0.201 (0.067)	0.032 (0.773)	−0.122 (0.269)	−0.037 (0.741)	0.253 * (0.020)
Annual max temp.	−0.363 ** (0.001)	−0.219 * (0.045)	−0.306 ** (0.005)	0.086 (0.438)	−0.221 * (0.043)	0.336 ** (0.002)
Annual min temp.	−0.198 (0.071)	−0.038 (0.732)	−0.150 (0.173)	−0.041 (0.710)	−0.083 (0.453)	0.348 ** (0.001)
Total precipitation	−0.060 (0.585)	0.050 (0.650)	−0.062 (0.575)	0.129 (0.241)	−0.078 (0.480)	0.235 * (0.031)
Altitude	−0.325 ** (0.003)	−0.412 ** (0.000)	−0.245 * (0.024)	0.279 * (0.010)	−0.126 (0.252)	−0.119 (0.281)

^a^ Correlation coefficient (*r*) written is significantly correlated between the variables compared. Positive values denote positive correlation, and negative values denote negative correlation. Values in brackets indicate *p* value (** *p* < 0.01, * *p* < 0.05).

**Table 9:** Pearson’s correlation coefficient between growth characteristics and active compounds of *Cudrania tricuspidata*.

	Correlation Coefficient (*r*) ^a^					
	Length of Fruit	Width of Fruit	Fresh Weight of Fruit	Sugar Contents of Fruit	Number of Seeds	Aspect Ratio
6,8-diprenylorobol	−0.436 ** (0.000)	−0.327 ** (0.002)	−0.447 ** (0.000)	0.310 ** (0.004)	−0.182 (0.098)	0.296 ** (0.006)
6,8-diprenylgenistein	−0.401 ** (0.000)	−0.351 ** (0.001)	−0.418 ** (0.000)	0.281 ** (0.010)	−0.217 * (0.047)	0.183 (0.095)
4′-*O*-methylalpinumisoflavone	−0.004 (0.972)	0.007 (0.951)	0.062 (0.577)	−0.118 (0.285)	−0.020 (0.853)	0.027 (0.806)
Total	−0.326 ** (0.002)	−0.267 * (0.014)	−0.301 ** (0.005)	0.161 (0.142)	−0.174 (0.113)	0.186 (0.090)

^a^ Correlation coefficient (*r*) written is significantly correlated between the variables compared. Positive values denote positive correlation, and negative values denote negative correlation. Values in brackets indicate *p* value (** *p* < 0.01, * *p* < 0.05).

## Data Availability

The data presented in this study are available under permission from the corresponding author on reasonable request.

## Supplementary Materials

The following supporting information can be downloaded at: https://www.mdpi.com/article/10.3390/plants12112107/s1, Table S1: Meteorological data of 28 different *Cudrania tricuspidata* cultivation sites; Table S2: Pearson’s correlation between growth characteristics of *Cudrania tricuspidata*; Table S3: Pearson’s correlation between soil properties of *Cudrania tricuspidata*; Table S4: Geographic information about the cultivation sites where fruits of *Cudrania tricuspidata* were collected in Republic of Korea.
