# Supplementary material for: The Growth Characteristics and the Active Compounds of Cudrania tricuspidata Fruits in Different Cultivation Environments in South Korea
Source: Plants (Basel). 2023 May 25;12(11):2107. doi: 10.3390/plants12112107 (PMC10255359; doi:10.3390/plants12112107)
Supplement: Supplementary file 1 [file plants-12-02107-s001.zip › plants-2403777-supplementary.pdf]

SUPPLEMENTARY DATA

Table S1. Meteorological data of 28 different *Cudrania tricuspidata* cultivation sites.

Cultivation Sites	Annual Mean Temp. (°C)	Annual Mean Max Temp. (°C)	Annual Mean Min Temp. (°C)	Annual Max Temp. (°C)	Annual Min Temp. (°C)	Total Precipitation (mm)
1	11.4	17.6	6.3	36.8	-19.9	923.0
2	11.4	17.8	6.3	35.2	-18.9	1112.1
3	13.6	19.9	8.3	36.3	-14.5	1142.8
4	15.4	21.1	10.7	37.8	-10.9	1708.2
5	15.6	20.1	11.9	36.2	-10.9	1800.1
6	14.8	20.9	9.6	35.7	-13.3	1557.0
7	13.5	20.5	7.7	37.0	-16.8	1302.5
8	13.9	20.0	8.6	36.6	-12.8	1112.1
9	12.5	18.2	7.5	34.7	-16.7	1367.3
10	10.0	16.1	4.6	31.2	-19.2	1129.1
11	13.2	19.0	8.2	34.7	-16.3	1230.8
12	13.6	20.6	7.7	36.6	-17.2	1159.0
13	11.5	18.6	5.7	36.4	-17.7	987.7
14	13.5	18.9	8.8	34.8	-19.1	1104.6
15	14.3	19.9	9.4	34.6	-14.1	1339.8
16	15.0	20.5	10.0	35.1	-11.9	1708.3
17	14.0	19.4	9.4	35.5	-17.4	1202.6
18	14.0	19.8	9.1	35.3	-15.9	1146.0
19	13.9	19.7	8.7	34.8	-17.1	1387.9
20	14.2	20.5	9.1	35.1	-16.5	1132.0
21	14.0	19.6	9.1	35.2	-18.1	1174.6
22	12.6	19.0	7.1	34.9	-21.3	1250.5
23	13.6	19.8	8.4	35.2	-21.5	1232.6
24	13.8	19.2	9.1	34.8	-17.6	1354.6
25	11.8	18.0	6.3	33.5	-24.6	1453.5
26	13.0	19.4	7.5	36.1	-21.4	1093.5
27	12.5	18.5	7.2	34.6	-20.5	1058.9
28	12.4	18.4	7.1	34.9	-19.6	1365.5

Table S2. Pearson's correlation coefficient between growth characteristics of *Cudrania tricuspidata*.

	Correlation Coefficient (*r*) [a]					
	**Length of Fruit**	**Width of Fruit**	**Fresh Weight of Fruit**	**Sugar Contents of Fruit**	**Number of Seeds**	**Aspect Ratio**
Length of Fruit	-	0.895 ** (0.000)	0.900 ** (0.000)	−0.487 ** (0.000)	0.569 ** (0.000)	−0.420 ** (0.000)
Width of Fruit		-	0.893 ** (0.000)	−0.433 ** (0.000)	0.478 ** (0.000)	−0.022 (0.842)
Fresh Weight of Fruit			-	−0.425 ** (0.000)	0.561 ** (0.000)	−0.201 (0.067)
Sugar Contents of Fruit				-	−0.356 ** (0.001)	0.207 (0.059)
Number of Seeds					-	−0.305 ** (0.005)
Aspect Ratio						-

[a] Correlation coefficient (*r*) written is significantly correlated between the variables compared. Positive values denote positive correlation and negative values denote negative correlation. Values in bracket means *p* value (** $p < 0.01$, * $p < 0.05$).

Table S3. Pearson's correlation coefficient between soil properties of *Cudrania tricuspidata.*

	Correlation Coefficient (r) [a]										
	**pH**	**EC**	**OM**	**TN**	**Avail. P_2O_5**	**K^+**	**Ca^{2+}**	**Mg^{2+}**	**Na^+**	**CEC**	**BS**
pH	-	0.968 **	0.115	0.515 **	0.447 **	0.327 **	0.142	0.887 **	0.119	0.807 **	0.036
		(0.000)	(0.296)	(0.000)	(0.000)	(0.002)	(0.199)	(0.000)	(0.279)	(0.000)	(0.748)
EC		-	0.224 *	0.527 **	0.425 **	0.366 **	0.056	0.861 **	0.132	0.846 **	0.045
			(0.041)	(0.000)	(0.000)	(0.001)	(0.610)	(0.000)	(0.231)	(0.000)	(0.683)
OM			-	0.331**	−0.075	0.249 *	−0.315 **	0.100	0.011	0.280 **	−0.061
				(0.002)	(0.496)	(0.023)	(0.003)	(0.363)	(0.924)	(0.010)	(0.580)
TN				-	0.408 **	0.438 **	−0.072	0.620 **	0.345 **	0.641 **	0.204
					(0.000)	(0.000)	(0.515)	(0.000)	(0.001)	(0.000)	(0.063)
Avail. P_2O_5					-	0.565 **	0.195	0.587 **	0.772 **	0.560 **	0.841 **
						(0.000)	(0.076)	(0.000)	(0.000)	(0.000)	(0.000)
K^+						-	−0.118	0.395 **	0.480 **	0.437 **	0.648 **
							(0.284)	(0.000)	(0.000)	(0.000)	(0.000)
Ca^{2+}							-	0.160	0.105	−0.135	0.099
								(0.145)	(0.340)	(0.220)	(0.369)
Mg^{2+}								-	0.265 *	0.789 **	0.133
									(0.015)	(0.000)	(0.227)
Na^+									-	0.343 **	0.790 **
										(0.001)	(0.000)
CEC										-	0.257 *
											(0.018)
BS											-

[a] Correlation coefficient (r) written is significantly correlated between the variables compared. Positive values denote positive correlation and negative values denote negative correlation. Values in bracket means p value (** $p < 0.01$, * $p < 0.05$).

Table S4. Geographic information about the cultivation sites where fruits of *Cudrania tricuspidata* were collected in South Korea.

Cultivation Sites	Name of Sites	Altitude (m)	N (Latitude)	E (Longitude)
1	Inje-gun	582	37.977778	128.238194
2	Jeongseon-gun	414	37.487500	128.585833
3	Sancheong-gun	325	35.397750	127.833611
4	Yangsan-si	58	35.377778	128.910000
5	Tongyeong-si	55	34.888333	128.395556
6	Hadong-gun	175	35.009722	127.801389
7	Haman-gun	111	35.200278	128.431667
8	Gyeongju-si	179	35.933611	129.098333
9	Mungyeong-si	310	36.604722	127.961944
10	Bonghwa-gun	220	36.844333	128.890250
11	Sangju-si	360	36.592778	127.904444
12	Seongju-gun	212	35.838167	128.177778
13	Cheongsong-gun	431	36.220833	128.909167
14	Sejong-si	90	36.618611	127.272222
15	Goheung-gun	88	34.473889	127.173889
16	Boseong-gun	15	34.697222	127.183333
17	Yeonggwang-gun	31	35.302500	126.563917
18	Hampyeong-gun	34	35.089250	126.584167
19	Haenam-gun	75	34.391667	126.564444
20	Hwasun-gun	260	35.074444	127.089167
21	Gochang-gun	31	35.553056	126.739083
22	Muju-gun	343	35.989444	127.795222
23	Sunchang-gun	180	35.416306	127.103056
24	Jeongeup-si	220	35.573889	127.015278
25	Jinan-gun	332	35.787222	127.510556
26	Gongju-si	60	36.377778	127.169444
27	Cheonan-si	134	36.824722	127.262778
28	Chungju-si	131	37.098889	127.923611
